# Dynamic regulation of CD300b and CD300f on myeloid cells in oral immunotherapy

**DOI:** 10.1016/j.jacig.2026.100766

**Published:** 2026-07-24

**Authors:** Na'ama Epstein-Rigbi, Michal Itan, Shmulik Avlas, Eytan Damari, Liat Nachshon, Yael Koren, Michael Levy, Michael R. Goldberg, Arnon Elizur, Ariel Munitz

**Affiliations:** a The Institute of Allergy, Immunology and Pediatric Pulmonology, Shamir Medical Center, Be’er Ya’akov, Israel; b Pediatrics Department, Gray Faculty of Medical and Health Sciences, Tel Aviv University, Tel Aviv, Israel; c Allergy and Immunology Unit, Assuta Ashdod Medical Center, Ashdod, Israel; d Faculty of Health Science, Ben Gurion University, Be’er Sheva, Israel; e Department of Microbiology and Clinical Immunology, Gray Faculty of Medical and Health Sciences, Tel Aviv University, Tel Aviv, Israel; f Department of Pediatrics, Assuta Ashdod Medical Center, Ashdod, Israel

**Keywords:** Oral immunotherapy (OIT), IgE-mediated food allergy, CD300 receptors, myeloid cells, desensitization

## Abstract

**Background:**

Oral immunotherapy (OIT) induces desensitization in IgE-mediated food allergy, yet the role of myeloid cells in acquisition of tolerance is unclear. CD300 receptors regulate activation of myeloid cells, with CD300f acting as an inhibitory receptor and CD300b as an activating receptor.

**Objective:**

We sought to investigate whether the modulation of CD300 receptors on myeloid cells during OIT may reflect effector cell reprogramming and serve as biomarkers of treatment response.

**Methods:**

Thirty-five patients undergoing OIT were prospectively enrolled. Peripheral blood was collected at baseline and during updosing; 19 patients completed sampling on reaching maintenance. CD300b and CD300f expression in eosinophils, monocytes, and neutrophils was analyzed by flow cytometry. Allergen-specific IgE and IgG4 levels were measured by ImmunoCAP. Associations with clinical parameters were assessed using logistic regression.

**Results:**

Baseline CD300b was higher in patients with lower starting doses (*P* ≤ .05). CD300f expression was lower in those with atopic dermatitis or multiple food allergies (*P* ≤ .03). A significant downregulation of CD300b expression in the surface of eosinophils, monocytes, and neutrophils was noted early in treatment (*P* ≤ .05). Longitudinally, the expression of CD300f increased on eosinophils, whereas the expression of CD300b decreased on the surface of monocytes and neutrophils. Specific IgE reduction correlated with downregulation of CD300b expression in monocytes (*R* = 0.51, *P* = .02) and higher CD300f expression in eosinophils (*R* = −0.45, *P* = .05).

**Conclusions:**

Downregulation of CD300b and upregulation of CD300f during OIT suggests myeloid cell reprogramming toward a less inflammatory phenotype. These dynamic changes in expression suggest CD300b and CD300f as candidate biomarkers for understanding and monitoring OIT response.

## Graphical abstract

**Figure:**
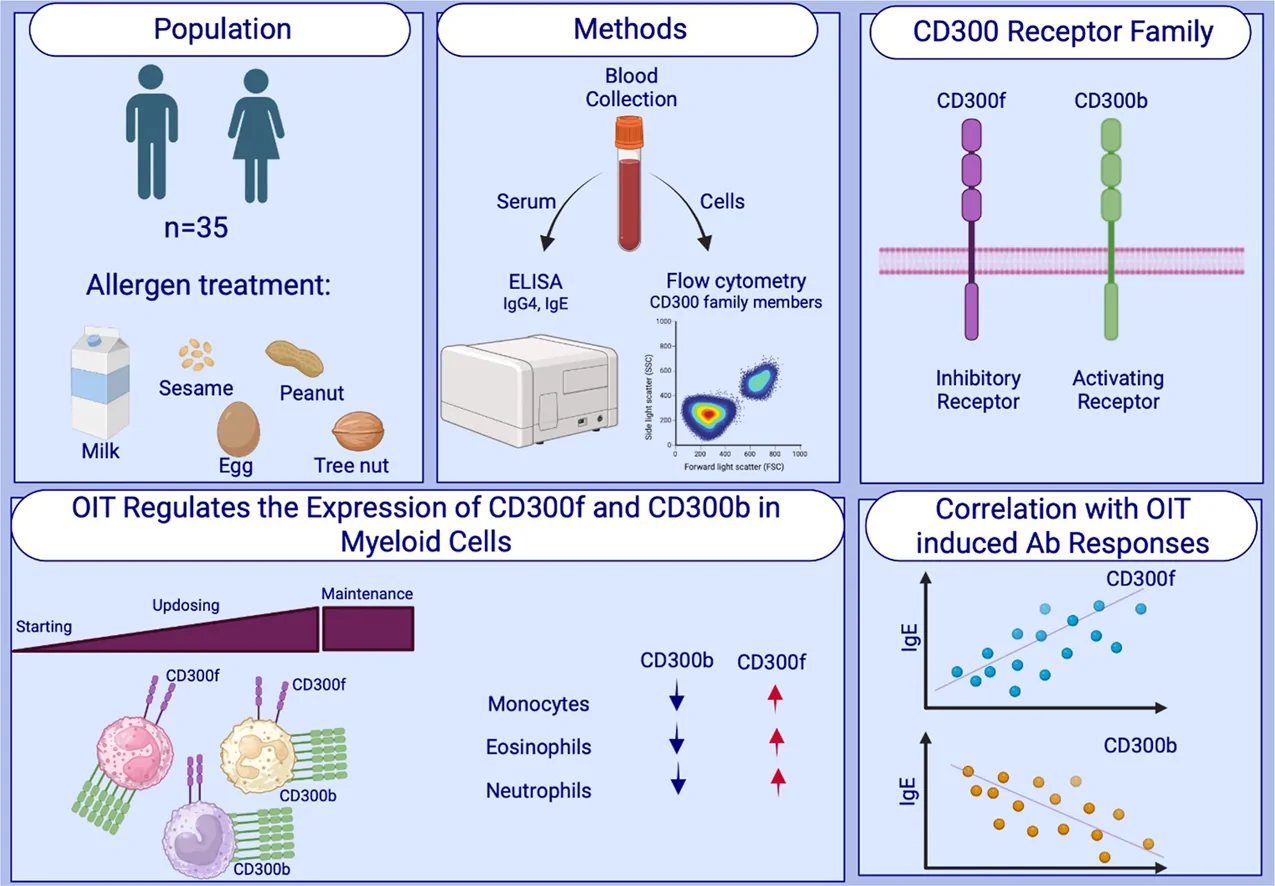

Oral immunotherapy (OIT) has emerged as a promising approach for the treatment of food allergy.^1^^,^^2^ Recent studies have demonstrated that OIT can induce desensitization and, in some patients, sustained unresponsiveness against the causative allergen.^3^ The underlying immunologic mechanisms driving this process have been primarily attributed to the induction of regulatory pathways, including increased frequencies of regulatory T cells and the upregulation of immunoregulatory cytokines such as IL-10 and TGF-β.^4^ However, the involvement of innate immune effector cells from the myeloid lineage including eosinophils, neutrophils, and monocytes remains poorly understood.^5^ Whether these innate immune cells are passively modulated by the tolerogenic immune environment or actively contribute to the induction and maintenance of tolerance remains an open question. This distinction is critical given the increasing appreciation of effector cell plasticity and their potential regulatory functions in allergic inflammation.^6^ A better understanding of the phenotypic and functional alterations in these cells during OIT may provide novel insights into the mechanisms governing tolerance.^7^

Members of the CD300 receptor family are expressed on multiple immune cell types, including eosinophils, neutrophils, monocytes, and mast cells.^8^ Among them, CD300a and CD300f are inhibitory receptors,^9^, ^10^, ^11^ whereas CD300b is an activating receptor.^12^^,^^13^ These receptors recognize lipid ligands such as phosphatidylserine and ceramide,^14^ and their expression has been associated with modulation of cell activation,^15^ cytokine release,^16^ and survival,^17^ particularly in the context of type 2 immune responses.^18^ Importantly, because CD300 receptors function as lipid-sensing receptors, alterations in lipid exposure and membrane composition during allergic inflammation or immunotherapy may directly influence CD300-mediated signaling and downstream effector cell responses. In eosinophils, CD300f engagement attenuates degranulation and cytokine secretion,^15^^,^^19^ whereas in monocytes and neutrophils, CD300b has been linked to enhanced responses to microbial products.^12^^,^^13^^,^^20^

Expression of CD300 molecules is dynamically regulated by environmental stimuli and inflammatory mediators.^21^ Type 2–associated cytokines, most notably IL-5, IL-33, IL-4, and IL-13, have been shown to modulate CD300 expression, particularly on eosinophils.^8^^,^^22^ IL-5 promotes eosinophil differentiation and survival and induces CD300f expression.^23^ Similarly, IL-33 can enhance CD300b and CD300f expression in myeloid cells, potentially modifying their activation threshold.^23^ Furthermore, CD300f is required for optimal activation of mast cells and eosinophils by IL-4.^15^ These findings demonstrate that CD300 molecules are not only markers of cell activation states but also have functional roles in shaping effector responses during allergic inflammation.

Despite the emerging significance of CD300 receptors in allergy, their expression and functional relevance during immunotherapy and specifically OIT remain largely unknown.^24^ Herein, we examined the expression of CD300a, CD300b, and CD300f on peripheral blood eosinophils, neutrophils, and monocytes in patients undergoing OIT for food allergy. We aimed to determine whether OIT alters the expression of these receptors on effector cells and whether such changes may reflect active immune modulation associated with tolerance acquisition. This study provides insight into the potential role of effector cells and their inhibitory or activating receptors in OIT and may uncover biomarkers indicative of treatment response and immunologic remodeling.

## Methods

### Study design and participants

Patients undergoing OIT for milk, egg, peanut, sesame, or tree nut allergy in the Shamir Medical Center, Israel, between March 2021 and July 2023 were recruited prospectively. All patients were recruited before starting the OIT program. The study was approved by the hospital ethical committee. All adult patients or caregivers for pediatric patients signed informed consent.

### The OIT program

The OIT program is individualized, performed in an ambulatory care setting. Patients older than 4 years are eligible to participate. IgE-mediated food allergy was defined by evidence of IgE sensitization, by either skin prick test or specific IgE, together with a positive oral food challenge, or a clinical history of an allergic reaction following accidental ingestion in the year previous to the start of OIT. Patients with a history of anaphylaxis and asthma were not excluded from treatment but asthma had to be well controlled throughout treatment. The program began with a 2- to 3-day dose-escalation phase during which the dose eliciting a reaction in each patient was established, and the starting dose determined. Dose escalation during the second and third rounds was limited to a maximum of 4-fold. Treatment subsequently consisted of alternate cycles of updosing and home-treatment phases until the full desensitization target dose (7200, 3000, 4000, and 6000 mg for milk, peanut, sesame and tree nut, and egg protein, respectively) was tolerated. Patients unable to reach this dose but able to consume a minimal dose of at least 300 mg of the allergen protein were defined as partially desensitized. Patients who stopped treatment altogether were considered as treatment failure. A similar treatment protocol was used for patients of all ages.

### Blood sample collection and processing

Blood was drawn at 3 time points: during the first induction days of OIT treatment representing baseline expression, during the first 3 months of updosing, and on finishing the updosing phase and reaching the maintenance dose. For 35 patients, blood was obtained at baseline and during the first phase of updosing. Only 19 of these patients completed the updosing phase within the time limits of the study and therefore blood was drawn for them in the 3 intended time points.

### Blood work analysis and flow cytometry

Identification of peripheral blood eosinophils, monocytes, and neutrophils was performed using flow cytometry. Peripheral blood leukocytes were analyzed directly following red blood cell lysis. Eosinophils were identified as CD45^+^Siglec-8^+^CD16^−^ cells, monocytes as CD45^+^CD14^+^ cells, and neutrophils as CD45^+^CD16^+^Siglec-8^−^ cells based on sequential gating strategies (see Fig E1 in this article’s Online Repository at www.jaci-global.org). The following antibodies were used: anti-CD16, anti–Siglec-8 (Biolegend, San Diego, Calif), anti-CD14 and anti-CD45 (Biogems, Westlake Village, Calif), and anti-CD300a, anti-CD300b, anti-CD300e, anti-CD300f, and anti-CD300c (R&D Systems, Minneapolis, Minn). Isotype controls including Rat IgG2A and Rat IgG2B were obtained from R&D Systems. Secondary antibodies including Donkey anti-Goat AlexaFlour 647, Goat anti-mouse AlexaFlour 488, and goat anti-rabbit AlexaFlour 647 were purchased from Jackson Immunoresearch (West Grove, Pa). At least 10,000 events were acquired and CD300 receptor expression was analyzed using Kaluze software (Becman Coulter, Indianapolis, Ind) as described.^15^ Briefly, antibody staining was compared to isotype control and mean fluorescent intensity (MFI) fold increase (FI) was calculated.

### Analysis of IgE and IgG4

Analysis of allergen-specific IgE and IgG4 in the serum of patients was conducted on samples that were obtained from each patient at baseline, early updosing phase, and after the completion of updosing and reaching maintenance. For each patient, measurements of allergen-specific IgE and allergen-specific IgG4 for the patient’s specific allergen treated in OIT were measured using ImmunoCAP (Thermo Fisher Scientific, Uppsala, Sweden).

### Statistical analysis

Statistical analysis aimed to compare CD300 receptor expression levels over time and to evaluate differences between patients who completed OIT updosing and maintenance versus those who did not finish updosing.

Normality of expression measures was assessed using the Shapiro-Wilk test. Because distributions deviated from normality, continuous variables were summarized using median and interquartile range (IQR), and categorical variables were described as counts and percentages. Nonparametric tests were used for group comparisons and for longitudinal evaluation.

Differences between groups were evaluated using the Mann-Whitney *U* test for continuous variables and the Fisher exact test for categorical variables. Subgroup analyses, including comparisons of patients with atopic dermatitis (AD) and those with multiple food allergies, were also performed using the Mann-Whitney *U* test.

Longitudinal changes in receptor expression across time points (baseline, updosing, and maintenance) were assessed using the Friedman test for repeated measures. Violin plots were used to illustrate changes in receptor expression over time.

Associations of CD300 receptor expression with serological markers of immune modulation (allergen-specific IgE and IgG4) were analyzed using Spearman rank correlation coefficients (*R*), with results visualized using scatterplots and fitted trendlines.

The sample size was determined by the number of patients recruited during the study period and was not powered for formal hypothesis testing. Missing data were not imputed, and analyses were based on available data only.

A 2-sided *P* value less than .05 was considered statistically significant. All analyses were conducted using R version 4.3.2 (R Foundation for Statistical Computing, Vienna, Austria).

## Results

### Patient demographic and clinical characteristics

A total of 35 patients were recruited. A schematic overview of patient enrollment, longitudinal sampling, treatment completion, partial desensitization, and study dropouts is shown in Fig E2 (in the Online Repository available at www.jaci-global.org). All 35 completed blood work before the start of OIT and during the first 3 months of updosing. Nineteen patients completed the full updosing period and were able to reach full desensitization within the study period. Among the remaining 16 patients, 5 dropped out, 1 because of relapse of eosinophilic esophagitis and the rest because of emotional difficulties. A subanalysis comparing clinical and demographic data between the patients who completed updosing (n = 19) and those who did not (n = 16) revealed a similar background (see Table E1 in this article’s Online Repository at www.jaci-global.org). The median age was 13 years (IQR, 9-19), and 19 patients (54.3%) were female. Most patients had an atopic background, 18 patients (51.4%) had multiple food allergies, 18 (51.4%) had asthma, 24 (68.6%) had house dust mite sensitivity, and 17 (48.6%) had AD. Clinically, most patients had had a prior anaphylactic reaction (62.9%), with 17 (48.6%) requiring epinephrine injection. Four patients (11.4%) had undergone prior OIT for a different food allergen (Table I).

**Table I:** Patient demographic and clinical data

Category	Parameter		Patients (n = 35)
Demographics	Age (y)		13 (9-19)
	Sex: female		19 (54.3)
Atopic background	Multiple food allergy		18 (51.4)
	Asthma		18 (51.4)
	HDM sensitivity		24 (68.6)
	AD		17 (48.6)
Clinical background	Prior anaphylaxis		22 (62.9)
	Prior epinephrine injection		17 (48.6)
	Prior OIT treatment (different allergen)		4 (11.4)
OIT	Allergen treated	Milk	9 (25.7)
		Peanut	10 (28.6)
		Sesame	8 (22.9)
		Walnut	6 (17.1)
		Egg	2 (5.7)
	Skin prick test wheal diameter (mm)		7 (5-9)
	Starting dose (mg protein)		20 (5-40)
	Interval first to second sample (mo)		1 (1-3)
	Duration of updosing (mo) (n = 19)		14 (11-16)

Categorical variables presented as numbers and percentage, continuous variables presented as median and interquartile ranges.

*HDM*, House dust mite.

Patients underwent OIT for milk (25.7%), peanut (28.6%), sesame (22.9%), walnut (17.1%), and egg (5.7%). The median skin prick test wheal diameter was 7 mm (IQR, 5-9). The median starting dose was 20 mg of protein (IQR, 5-40) (Table I).

### Early OIT updosing is associated with downregulation of CD300b and mild CD300f upregulation

To explore whether OIT modulates CD300 receptor expression, we analyzed the expression of CD300a, CD300b, CD300c, CD300e, and CD300f on eosinophils, monocytes, and neutrophils in 35 patients at baseline and after the early updosing phase. A subanalysis comparing baseline expression levels in patients who eventually completed OIT (n = 19) versus those still updosing or who dropped out (n = 16) identified modest differences in several CD300 receptors, including higher baseline expression of CD300f on eosinophils and CD300b on monocytes in patients who completed OIT within the study time frame (see Table E2 in this article’s Online Repository at www.jaci-global.org). However, these findings were not uniformly observed across all receptors or cell populations.

**Fig 1:**
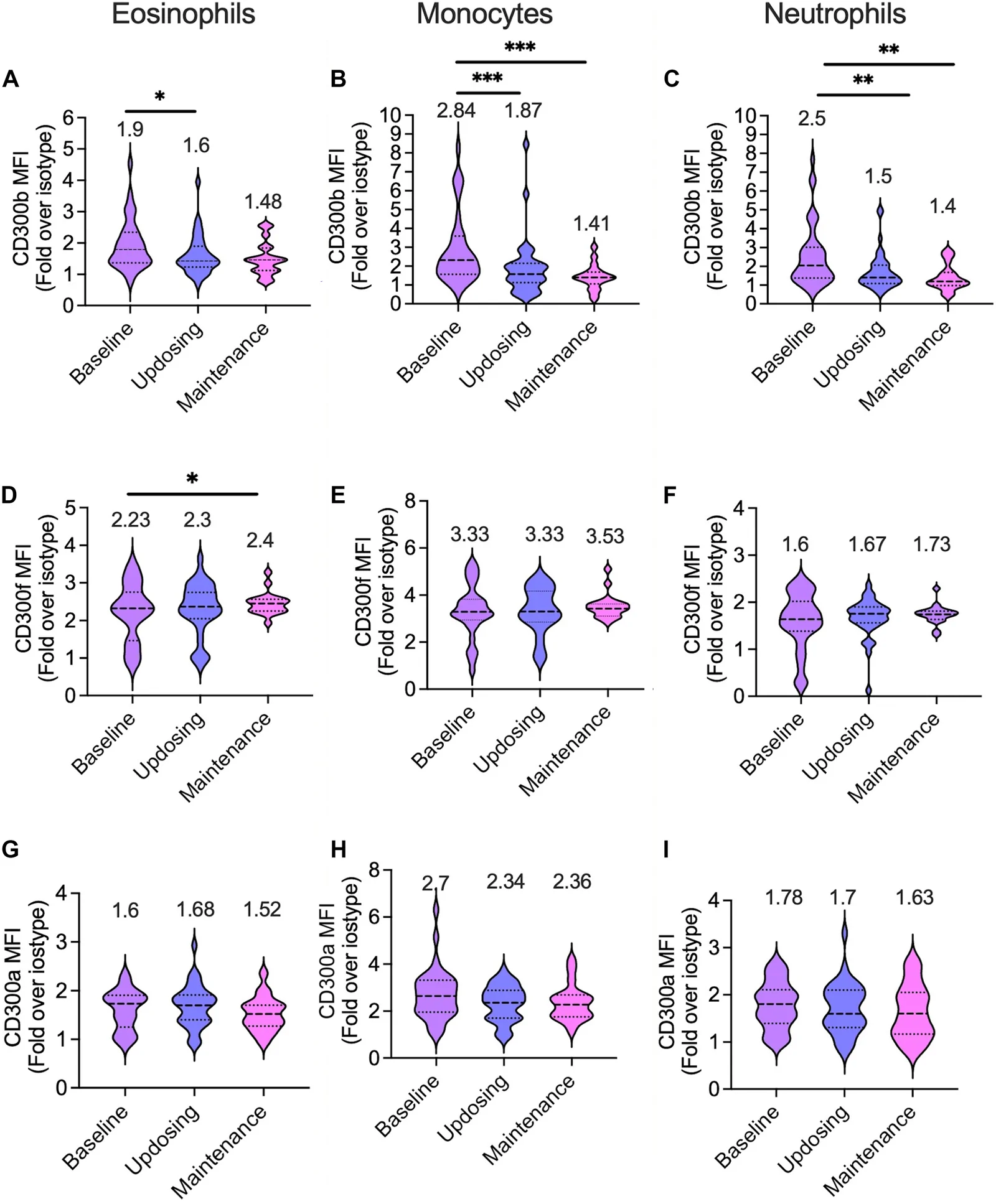
Early OIT updosing is associated with downregulation of CD300b on peripheral myeloid cells. Flow cytometric analysis of CD300 receptor expression in eosinophils (**A**, **D**, **G**), monocytes (**B**, **E**, **H**), and neutrophils (**C**, **F**, **I**) at baseline and during early updosing (n = 35). CD300b expression was significantly reduced across all 3 cell populations (Fig 1, *A-C*), whereas CD300a and CD300f expression remained largely unchanged (Fig 1, *D-I*). Data are presented as FI in MFI relative to isotype control. Paired comparisons were analyzed using the Wilcoxon signed-rank test (n = 35). ∗*P* < .05, ∗∗*P* < .001, ∗∗∗*P* < .0001.

During early updosing, CD300b expression was significantly reduced across all 3 myeloid populations. Specifically, expression in eosinophils declined from a median FI in MFI of 1.94 to 1.6 (*P* = .0437), on monocytes from 2.84 to 1.87 (*P* = .0004), and on neutrophils from 2.5 to 1.5 (*P* = .0085) (Fig 1, *A-C*). This consistent downregulation may reflect early shifts in effector cell phenotype during immune modulation.

Although CD300a and CD300f were readily detectable on the surface of eosinophils, monocytes, and neutrophils, their overall expression did not change (Fig 1, *B-I*).

CD300c and CD300e were not detected on the surface of eosinophils, or neutrophils, at either time point, with fluorescence intensities comparable to isotype controls (FI in MFI ∼1). Similarly, the expression of CD300c was undetected in monocytes. Nonetheless, CD300e was marginally expressed in monocytes under baseline conditions (FI in MFI 2.3) but its expression was not unchanged throughout the treatment regimen (see Tables E3 and E4 in this article’s Online Repository at www.jaci-global.org).

### CD300b and CD300f expression at baseline reflects starting dose, AD, and multiple food allergy

**Fig 2:**
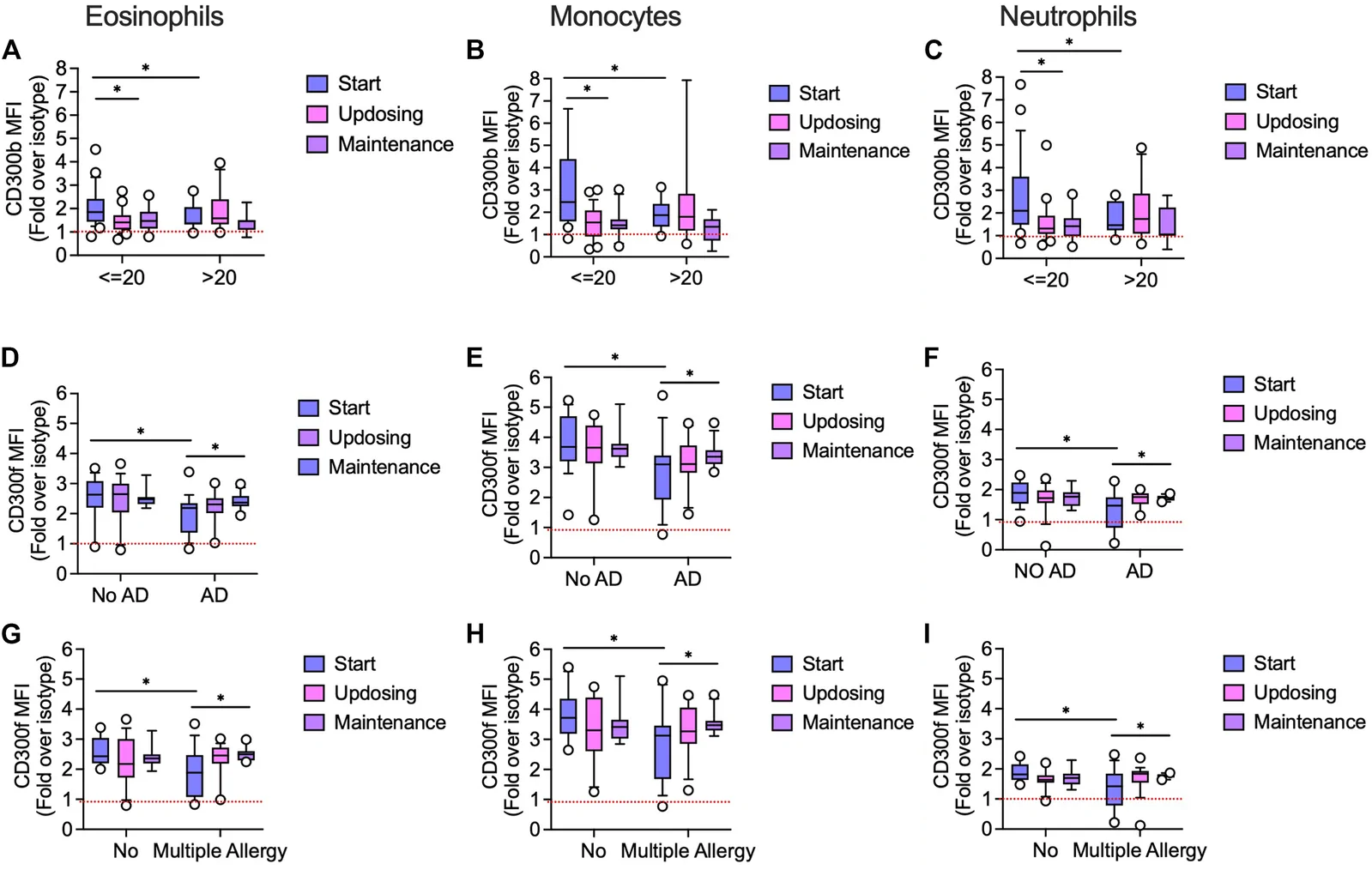
Baseline CD300b and CD300f expression is associated with OIT starting dose, AD, and multiple food allergy. Baseline CD300b expression in eosinophils, monocytes, and neutrophils stratified by starting dose (<20 mg vs ≥20 mg protein) (**A-C**). Baseline CD300f expression stratified by presence of AD (**D-F**) and multiple food allergy (**G-I**). Longitudinal changes demonstrate increased CD300f expression during OIT in patients with AD or multiple food allergies. Data are presented as median with IQR. Group comparisons were performed using the Mann-Whitney *U* test (n = 35). ∗*P* < .05.

Next, we examined whether baseline receptor expression is associated with any clinical parameters or treatment protocols. Baseline CD300b expression was significantly higher in patients who began OIT with a starting dose less than 20 mg protein (n = 12) compared with those who started at 20 mg or more (n = 7). For example, in eosinophils, the median FI of CD300b MFI was 1.85 versus 1.37 (*P* = .05); in monocytes, the median FI of CD300b MFI was 2.46 versus 1.86 (*P* = .001); and in neutrophils, the median FI of CD300b MFI was 2.1 vs. 1.63 (*P* = .005) (Fig 2, *A-C*). Notably, the expression of CD300b was decreased following OIT treatment only in the patient group whose starting dose was 20 mg or less (Fig 2, *A-C*).

In contrast to CD300b, baseline expression of CD300f was lower on the surface of myeloid cells obtained from patients with AD (n = 11) and from patients with multiple food allergies (n = 10) (Fig 2, *D-I*). For instance, in eosinophils, the median FI of CD300f MFI was 2.18 in patients with AD versus 2.63 in patients without AD (*P* = .02); in monocytes, the median FI of CD300f MFI was 3.1 in patients with AD versus 3.68 in patients without AD (*P* = .01); and in neutrophils, the median FI of CD300f MFI was 1.46 in patients with AD versus 1.89 in patients without AD (*P* = 0.03) (Fig 2, *D-F*). Similarly, patients with multiple food allergies (n = 10) had lower baseline CD300f expression compared with those allergic to a single food (n = 9, 1.88 vs 2.43 in eosinophils [*P* = .02]; 3.12 vs 3.71 in monocytes [*P* < .01]; and 1.42 vs 1.81 in neutrophils (*P* < .01]) (Fig 2, *G-I*). Importantly, assessment of CD300f expression during OIT treatment revealed that the expression of CD300f was increased during OIT treatment (from start to maintenance) in eosinophils, monocytes, and neutrophils of patients with AD or multiple food allergies (Fig 2, *D-I*).

Other clinical parameters including age, sex, skin prick test wheal size, asthma status, prior OIT exposure, baseline absolute eosinophil count, multiple food allergy, and the specific allergen used for OIT were not associated with differences in CD300 expression patterns.

### Longitudinal changes in CD300f and CD300b during OIT updosing

Nineteen patients completed longitudinal profiling of CD300 receptor expression across 3 time points (ie, baseline, updosing, and maintenance). Among the receptors analyzed, dynamic changes were observed in CD300b and CD300f in several cell types over the course of OIT updosing.

Expression of CD300b in monocytes markedly declined over time, from a fold expression of 2.84 MFI at baseline to 1.41 MFI at the end of updosing (*P* < .001). Similarly, the expression of CD300b in neutrophils decreased from a baseline fold expression of 2.5 MFI to 1.4 MFI (*P* = .006) (Fig 1, *B* and *C*).

Expression of CD300f in eosinophils increased significantly from baseline to the end of updosing (2.22 to 2.4, *P* = .048), suggesting gradual upregulation during treatment (Fig 1, *D*).

No additional longitudinal changes were detected in the expression of other CD300 family members on eosinophils, monocytes, or neutrophils during the same period.

### CD300 expression dynamics correlate with allergen-specific IgE and IgG4 responses during OIT

**Fig 3:**
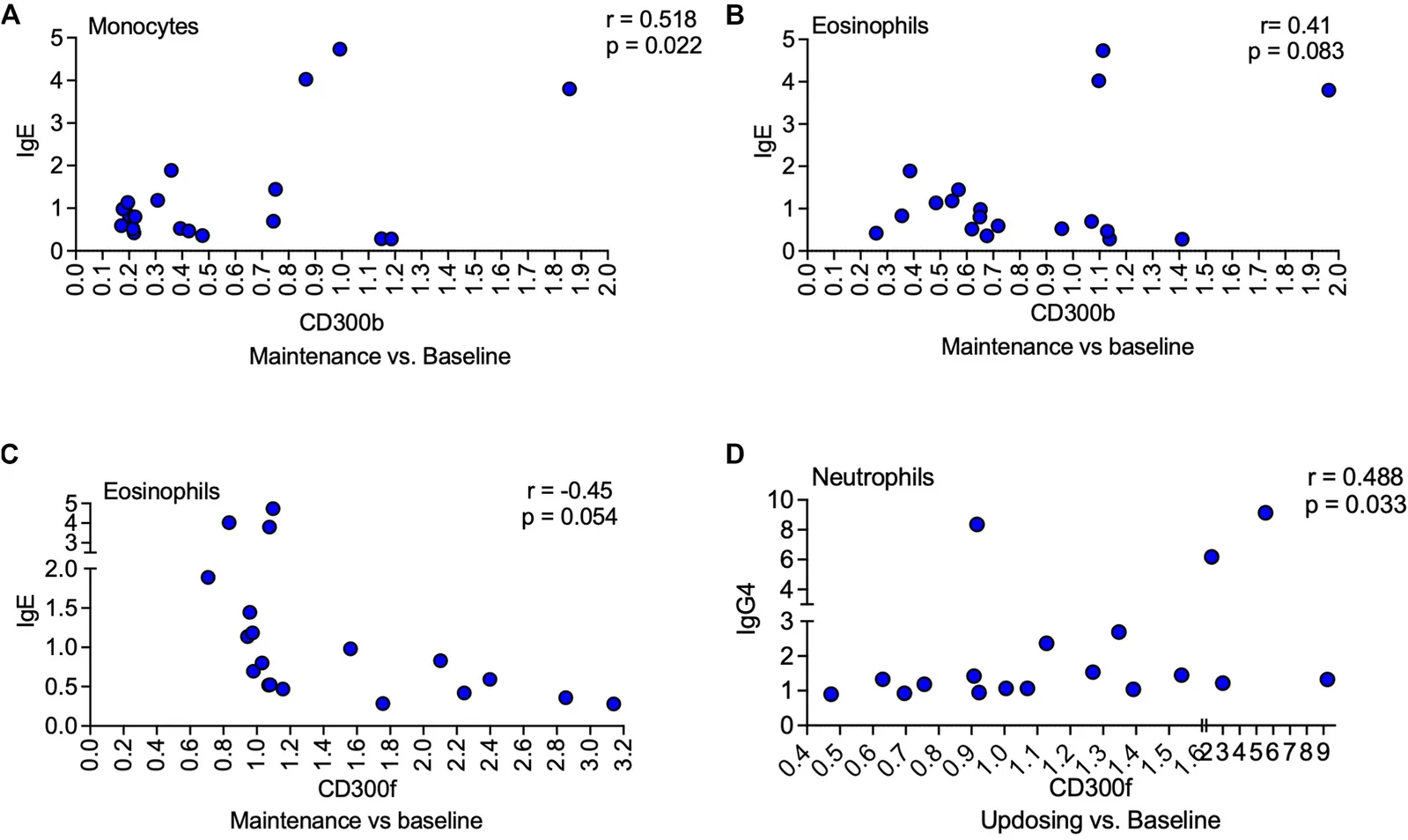
Changes in CD300 receptor expression correlate with allergen-specific IgE and IgG4 responses during OIT. Reduction in allergen-specific IgE from baseline to maintenance correlates with decreased CD300b expression on monocytes (**A**) and eosinophils (**B**). Higher CD300f expression on eosinophils correlates with greater IgE reduction (**C**), whereas increased CD300f expression on neutrophils correlates with increased allergen-specific IgG4 levels (**D**). Associations were assessed using Spearman rank correlation (n = 19).

We further examined whether changes in CD300 receptor expression were associated with serological markers of immune modulation during OIT (n = 19). Reduction in allergen-specific IgE between baseline and maintenance was associated with a larger decrease in CD300b expression in monocytes (*R* = 0.51, *P* = .022) (Fig 3, *A*). A similar trend was observed in eosinophils as well (*R* = 0.41, *P* = .083) (Fig 3, *B*). No association was found between the decrease in CD300b expression and the increase in allergen-specific IgG4 levels over the course of treatment.

Patients who exhibited higher expression of CD300f in their eosinophils throughout OIT were associated with a more pronounced decrease in allergen-specific IgE (*R* = −0.45, *P* = .054) (Fig 3, *C*). This association was not observed in monocytes or neutrophils (not shown). In contrast, neutrophils with elevated CD300f expression between baseline and early updosing showed a positive correlation with increased allergen-specific IgG4 (*R* = 0.48, *P* = .03) (Fig 3, *D*). No additional associations were observed in other cell types or at different time points (not shown).

## Discussion

OIT has been shown to induce clinical desensitization and, in a subset of patients, sustained immune tolerance in IgE-mediated food allergy.^25^ Although adaptive immune mechanisms, particularly the induction of regulatory T cells and immunoregulatory cytokines, have been extensively studied,^4^^,^^26^ the contribution of innate immune effector cells to this process remains incompletely defined. In Israel, OIT is primarily conducted in specialized tertiary-care allergy centers, including the center in which this study was performed. In this study, we examined the dynamic regulation of CD300 family receptors on peripheral myeloid cells during OIT and demonstrate that treatment is associated with coordinated modulation of activating and inhibitory signaling pathways in eosinophils, monocytes, and neutrophils. These findings suggest that desensitization during OIT extends beyond adaptive regulation and involves reprogramming of myeloid innate effector cell phenotypes.

The most consistent and robust observation in our study was the progressive downregulation of the activating receptor CD300b, particularly on monocytes, observed both early during updosing and longitudinally throughout OIT. CD300b is an immunoreceptor known to amplify inflammatory responses through cooperation with pattern-recognition receptors such as TLR4 and through recognition of phosphatidylserine on apoptotic cells and stressed tissues.^12^^,^^13^^,^^20^^,^^26^ Its downregulation during OIT suggests attenuation of monocyte activation potential and may reflect a shift toward a more restrained innate immune state. This interpretation aligns with emerging models of immune tolerance in which suppression of innate effector activation accompanies adaptive immune regulation.^27^^,^^28^ Importantly, baseline CD300b expression was higher in patients requiring lower starting doses, and its subsequent decline correlated with reductions in allergen-specific IgE, supporting its relevance not only as a mechanistic marker but also as a potential indicator of treatment trajectory.

In parallel with CD300b downregulation, we observed a modest but significant increase in the expression of the inhibitory receptor CD300f, most prominently on eosinophils during longitudinal follow-up. CD300f is a well-characterized inhibitory receptor that regulates eosinophil survival, cytokine secretion, and responsiveness to type 2 cytokines.^9^^,^^11^^,^^19^^,^^28^ Engagement of CD300f has been shown to dampen eosinophil effector functions and to restrain allergic inflammation in both human and murine systems.^15^^,^^19^^,^^23^ In our cohort, higher CD300f expression on eosinophils was associated with greater reductions in allergen-specific IgE, suggesting that enhanced inhibitory signaling in eosinophils accompanies successful immune modulation during OIT. These findings are consistent with experimental models demonstrating that CD300f agonism can suppress food-allergic responses and anaphylaxis,^29^ and further support a functional role for eosinophil inhibitory pathways in induction of tolerance.

Baseline CD300f expression was lower in patients with AD and in those with multiple food allergies. This is important because these 2 clinical phenotypes are associated with more severe or persistent allergic disease.^15^ Reduced expression of inhibitory receptors in these populations may reflect a heightened baseline activation state of myeloid cells, potentially contributing to disease chronicity. Notably, CD300f expression increased during OIT in these subgroups, suggesting that immunotherapy can partially reverse this inhibitory deficit. These findings support the concept that OIT not only suppresses allergen-specific adaptive responses but also recalibrates innate immune inhibitory pathways in patients with more complex atopic disease.

The reciprocal regulation that we observed (ie, downregulation of the activating receptor CD300b alongside upregulation of the inhibitory receptor CD300f across distinct myeloid subsets) suggests a coordinated mechanism of immune restraint. Such bidirectional modulation may raise activation thresholds and limit effector responses during allergen exposure, thereby contributing to clinical desensitization.^29^ Beyond their activating or inhibitory properties, CD300 receptors are lipid-sensing receptors that recognize phospholipids exposed on activated, apoptotic, or stressed cells. Thus, the dynamic regulation of CD300b and CD300f during OIT may also reflect alterations in lipid-mediated signaling pathways that accompany repeated allergen exposure, tissue adaptation, and immune remodeling. Although lipid metabolism was not directly assessed in our study, these findings raise the possibility that changes in the local lipid microenvironment may contribute to modulation of myeloid cell activation thresholds during acquisition of tolerance. This pattern is consistent with previous work demonstrating cooperative and sometimes opposing functions among CD300 family members in regulating mast cell and eosinophil activation.^11^^,^^15^ Moreover, genetic studies in mice indicate that disruption of CD300 inhibitory pathways alters susceptibility to anaphylaxis,^11^ further underscoring the importance of balanced CD300 signaling in allergic responses.^11^

Beyond mechanistic insight, our findings highlight CD300b and CD300f as potential peripheral biomarkers of OIT response. CD300b expression reflected initial dosing sensitivity and tracked with IgE reduction, whereas CD300f expression correlated with favorable serologic outcomes, including IgE decline and IgG4 induction. Given that these changes were detectable in peripheral blood, monitoring CD300 receptor expression may provide a practical and accessible window into immunologic remodeling during OIT and could aid in patient stratification or treatment monitoring.

Several limitations of this study should be acknowledged. First, longitudinal analyses were limited to patients who completed all sampling time points within the study period, resulting in a modest sample size, which was relatively small to begin with. Although the observed trends were consistent and biologically plausible, larger cohorts will be required to validate these findings. In addition, independent validation in separate pediatric and adult OIT cohorts will be important to determine the reproducibility and generalizability of the observed CD300 receptor dynamic. Moreover, we did not compare the results to a control group with food allergy that did not undergo OIT. Second, our analyses were confined to peripheral blood, which may not fully capture local immune dynamics at mucosal sites such as the gastrointestinal tract or skin, where allergen exposure and tolerance induction occur.^30^^,^^31^ In addition, our study focused primarily on circulating myeloid effector cells and did not assess additional cell populations that may contribute to OIT-induced tolerance, including basophils, dendritic cells, or enterocytes. Although these cell types are highly relevant to food allergy pathogenesis and mucosal immune regulation, their analysis is technically challenging in the context of routine pediatric peripheral blood sampling or would require invasive tissue acquisition. Future studies integrating tissue-specific and additional innate immune populations will be important to further define the cellular networks underlying OIT-induced immune remodeling. Third, functional assays were not performed to directly assess how CD300 modulation alters myeloid cell behavior during OIT. Future studies using dedicated prospective cohorts and larger sampling volumes will be required to determine whether the observed changes in CD300 receptor expression are associated with altered effector functions including degranulation, cytokine secretion, and activation thresholds in eosinophils, neutrophils, and monocytes during OI. Although correlations with serologic markers support a functional role, causality remains to be established. Finally, given the inclusion of multiple allergens and individualized treatment protocols, future studies with larger allergen-specific cohorts will be required to determine whether distinct allergens induce differential CD300-associated immune remodeling or whether the observed changes represent a shared pathway of immune tolerance during OIT. In addition, correlations between CD300 receptor expression and allergen-specific IgE or IgG4 were based on longitudinal within-patient changes rather than direct comparison of absolute immunoglobulin levels across different allergens.

### Conclusion

This study demonstrates that OIT is associated with dynamic and coordinated modulation of CD300 receptor expression on peripheral myeloid cells. The observed attenuation of activating signals alongside enhancement of inhibitory pathways supports a model in which innate effector cell reprogramming accompanies and potentially reinforces adaptive immune tolerance during OIT. These findings expand the current understanding of OIT mechanisms and identify CD300b and CD300f as candidate biomarkers and regulatory nodes in allergen immunotherapy.

## Disclosure statement

A.M. is supported by the US-Israel Bi-national Science Foundation (grant no. 2023029), the 10.13039/501100003977Israel Science Foundation (grant no. 542/20), the Israel Cancer Research Fund, the Israel Cancer Association Avraham Rotstein Donation, the Cancer Biology Research Center (TAU), and the Azrieli Foundation Canada-Israel. M.R.G. is funded by a Kamea grant from the Ministry of Health, Israel. M.E.-R. received a research grant from the Israel Allergy, Asthma and Immunology Association.

Disclosure of potential conflict of interest: The authors declare that they have no relevant conflicts of interest.

## Supplementary data

Supplementary Fig E1

Supplementary Fig E2

Supplementary Tables E1-E4
