# Supplementary material for: Dynamic regulation of CD300b and CD300f on myeloid cells in oral immunotherapy
Source: J Allergy Clin Immunol Glob. 2026 Jul 24;5(6):100766. doi: 10.1016/j.jacig.2026.100766 (PMC13572044; doi:10.1016/j.jacig.2026.100766)

Figure 1s. Gating strategy for identifying myeloid cells and CD300 receptor expression

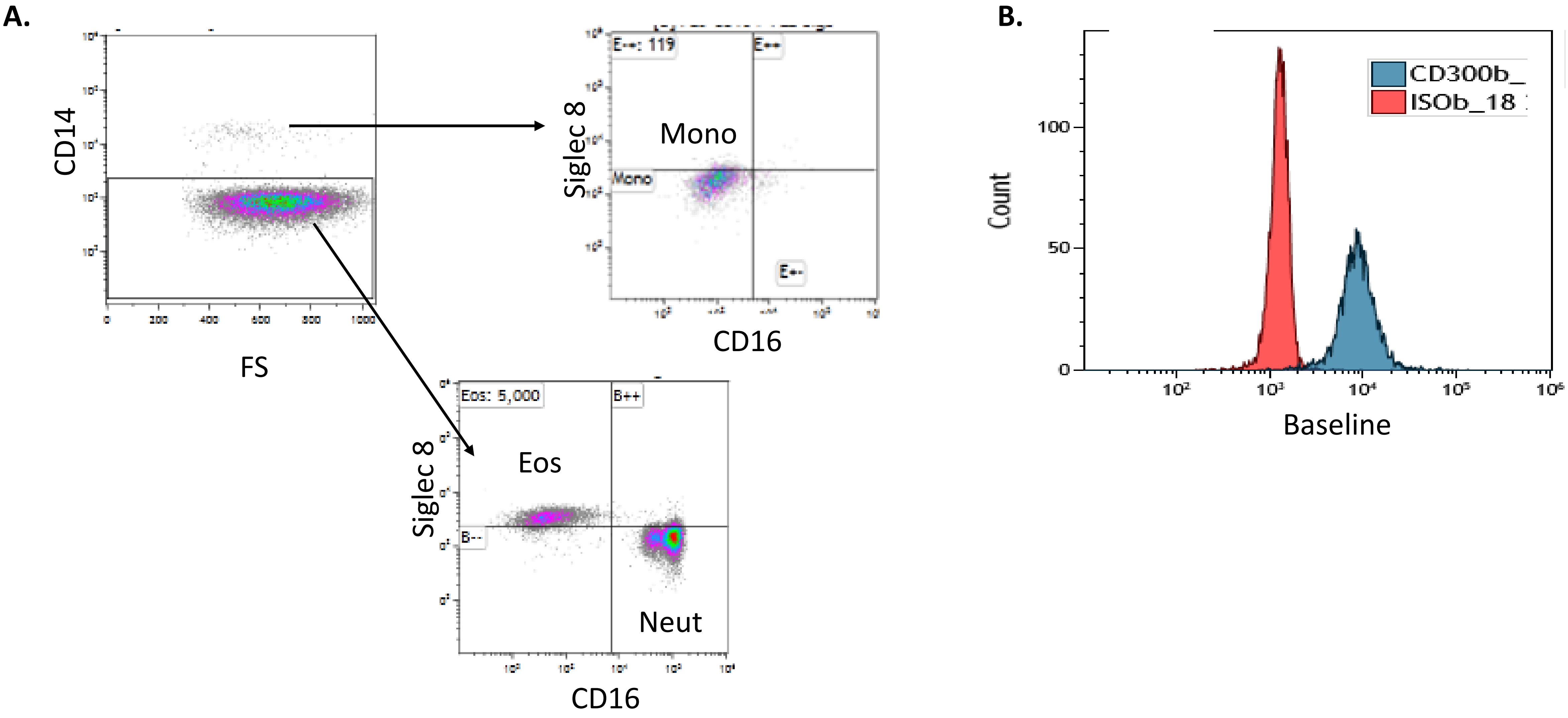

Supplement: Supplementary file 1 [file mmc1.pdf]
