# Supplementary material for: Dynamic regulation of CD300b and CD300f on myeloid cells in oral immunotherapy
Source: J Allergy Clin Immunol Glob. 2026 Jul 24;5(6):100766. doi: 10.1016/j.jacig.2026.100766 (PMC13572044; doi:10.1016/j.jacig.2026.100766)
Supplement: Supplementary file 3 [file mmc3.docx]

**Supplementary Table 1.** Comparison of clinical and demographic data between patients who completed up-dosing (n=19) to those that did not (n=16)

| **Category** | **Parameter** | **Completed up-dosing**  **n=19** | **Did not complete up-dosing n=16** | **P value** |
| --- | --- | --- | --- | --- |
| **Demographics** | Age | 11.0 (9.0–13.5) | 15.5 (8.5–21.5) | 0.435 |
|  | Female | 8 (42.1%) | 11 (68.8%) | 0.22 |
| **Atopic background** | Asthma | 11 (57.9%) | 7 (43.8%) | 0.62 |
|  | Atopic dermatitis | 11 (57.9%) | 6 (37.5%) | 0.315 |
|  | Allergic Rhinitis | 14 (73.7%) | 10 (62.5%) | 0.716 |
|  | Multiple food allergy | 10 (52.6%) | 8 (50%) | 1.0 |
|  | Prior Epinephrine | 9 (47.4%) | 8 (50%) | 1.0 |
|  | Prior Anaphylaxis | 13 (68.4%) | 9 (56.2%) | 0.503 |
| **Allergen treated** | Milk | 4 (21.1%) | 5 (31.2%) | 0.638 |
|  | Peanut | 2 (10.5%) | 0 (0.0%) |  |
|  | Sesame | 5 (26.3%) | 5 (31.2%) |  |
|  | Walnut | 4 (21.1%) | 4 (25.0%) |  |
|  | Egg | 4 (21.1%) | 2 (12.5%) |  |
| **Oral immunotherapy** | Prior OIT | 2 (10.5%) | 2 (12.5%) | 1.0 |
|  | SPT (mm wheal) | 6.0 (5.0–9.5) | 7.5 (6.0–9.0) | 0.677 |
|  | Starting dose^†^ | 20.0 (10.0–60.0) | 10.0 (5.0–25.0) | 0.228 |

Categorical variables presented as numbers and percentage, continuous variables presented as median and interquartile ranges.

^†^mg protein

**Supplementary Table 2.** Comparison between baseline CD300 expression of 19 patients who reached the end of up-dosing vs 16 who did not

| **Myeloid cell** | **Receptor** | **Patients that completed Up-dosing n=19** | **Patients that did not complete Up-dosing n=16** | **P value** |
| --- | --- | --- | --- | --- |
| **Eosinophils** | CD300a | 1.8 (1.4, 1.9) | 1.7 (1.4, 1.9) | 0.827 |
|  | CD300b | 1.9 (1.3, 2.9) | 1.7 (1.5, 2.2) | 0.743 |
|  | CD300c | 1.0 (0.9, 1.1) | 1.0 (0.9, 1.1) | 0.884 |
|  | CD300e | 1.0 (1.0, 1.1) | 1.1 (1.0, 1.2) | **0.038** |
|  | CD300f | 2.4 (2.3, 3.1) | 2.2 (1.4, 2.4) | **0.018** |
| **Monocytes** | CD300a | 2.8 (1.9, 3.3) | 2.5 (2.0, 3.1) | 0.855 |
|  | CD300b | 2.7 (1.9, 5.2) | 1.9 (1.6, 2.4) | **0.045** |
|  | CD300c | 1.3 (1.0, 1.9) | 1.5 (1.1, 1.9) | 0.584 |
|  | CD300e | 2.2 (1.8, 2.4) | 2.4 (1.8, 2.7) | 0.308 |
|  | CD300f | 3.2 (2.4, 3.5) | 3.6 (3.2, 4.3) | 0.068 |
| **Neutrophils** | CD300a | 1.7 (1.4, 2.0) | 1.9 (1.4, 2.3) | 0.343 |
|  | CD300b | 2.6 (1.7, 3.4) | 1.7 (1.3, 2.1) | 0.074 |
|  | CD300c | 1.1 (0.8, 1.2) | 1.0 (0.8, 1.2) | 0.560 |
|  | CD300e | 1.0 (0.9, 1.1) | 1.1 (1.0, 1.2) | **0.031** |
|  | CD300f | 1.7 (1.5, 2.3) | 1.5 (1.0, 1.8) | 0.117 |

continuous variables presented as median and interquartile ranges.

**Supplementary Table 3.** CD300c expression on myeloid cells

| **Study No** | **Baseline** | | | **Up-dosing** | | | **Maintenance** | | |
| --- | --- | --- | --- | --- | --- | --- | --- | --- | --- |
|  | **Eosinophils** | **Monocytes** | **Neutrophils** | **Eosinophils** | **Monocytes** | **Neutrophils** | **Eosinophils** | **Monocytes** | **Neutrophils** |
| **1** | 1.129 | 3.555 | 1.372 | 1.050 | 2.213 | 1.173 | 1.051 | 1.658 | 0.969 |
| **5** | 1.095 | 1.406 | 1.078 | 1.023 | 1.684 | 0.985 | 1.412 | 1.835 | 1.514 |
| **6** | 0.827 | 1.107 | 0.595 | 1.068 | 1.780 | 1.363 | 0.736 | 1.039 | 0.774 |
| **7** | 1.198 | 2.752 | 1.380 | 1.234 | 2.791 | 1.435 | 1.006 | 2.118 | 1.277 |
| **8** | 1.252 | 3.904 | 1.332 | 0.748 | 0.806 | 0.735 |  | | |
| **9** | 0.859 | 1.155 | 0.717 | 0.946 | 1.061 | 0.983 | 0.689 | 0.871 | 0.664 |
| **10** | 0.864 | 0.921 | 0.918 | 0.575 | 1.072 | 0.247 | 1.008 | 1.296 | 1.048 |
| **17** | 1.195 | 2.352 | 1.461 | 1.085 | 3.319 | 1.195 | 1.125 | 1.473 | 1.258 |
| **18** | 1.055 | 1.512 | 1.238 | 1.001 | 1.384 | 1.113 | 1.180 | 1.672 | 1.300 |
| **19** | 0.975 | 1.426 | 0.984 | 0.884 | 1.139 | 0.891 | 1.095 | 1.845 | 1.213 |
| **20** | 0.738 | 1.022 | 0.792 | 0.782 | 1.061 | 0.811 | 1.190 | 2.442 | 1.361 |
| **21** | 1.070 | 0.052 | 1.071 | 1.077 | 1.935 | 1.142 | 1.168 | 12.855 | 1.851 |
| **22** | 1.052 | 0.046 | 0.941 | 1.013 | 1.842 | 1.140 | 0.819 | 1.634 | 1.484 |
| **23** | 1.072 | 2.139 | 1.034 | 1.025 | 1.612 | 1.068 | 1.055 | 1.629 | 1.076 |
| **24** | 1.075 | 1.139 | 1.127 | 1.032 | 1.151 | 1.116 |  | | |
| **25** | 1.154 | 2.033 | 1.276 | 1.059 | 1.816 | 1.118 |  |  |  |
| **26** | 0.724 | 0.920 | 0.501 | 0.689 | 0.991 | 0.562 |  |  |  |
| **27** | 1.302 | 4.459 | 1.572 | 1.026 | 1.894 | 1.044 |  |  |  |
| **32** | 1.069 | 1.512 | 1.127 | 0.808 | 0.724 | 0.788 |  |  |  |
| **33** | 0.954 | 1.530 | 0.971 | 0.979 | 1.327 | 1.060 |  |  |  |
| **34** | 0.637 | 0.720 | 0.483 | 0.810 | 0.590 | 0.787 |  |  |  |
| **35** | 1.297 | 2.364 | 1.346 | 1.202 | 1.934 | 1.360 | 0.983 | 1.748 | 0.773 |
| **36** | 0.897 | 1.616 | 0.766 | 0.546 | 0.648 | 0.449 | 1.307 | 3.137 | 1.507 |
| **37** | 0.969 | 1.777 | 1.102 | 0.958 | 1.499 | 0.913 |  | | |
| **38** | 0.589 | 0.880 | 0.634 | 1.107 | 2.003 | 1.085 |  |  |  |
| **39** | 1.029 | 1.588 | 1.576 | 0.989 | 1.242 | 0.987 |  |  |  |
| **40** | 1.033 | 1.139 | 1.122 | 1.328 | 1.700 | 1.689 |  |  |  |
| **41** | 1.002 | 1.000 | 1.048 | 0.919 | 1.414 | 1.006 | 1.170 | 1.798 | 1.586 |
| **42** | 0.924 | 1.131 | 0.893 | 0.977 | 1.320 | 1.036 | 0.992 | 1.364 |  |
| **43** | 0.865 | 1.888 | 0.833 | 1.091 | 1.724 | 1.102 |  | | |
| **44** | 0.747 | 1.322 | 0.614 | 0.886 | 2.113 | 1.012 | 0.808 | 0.972 | 0.779 |
| **45** | 1.004 | 1.288 | 1.075 | 0.939 | 1.453 | 0.970 | 0.892 | 1.735 | 1.113 |
| **46** | 0.474 | 0.578 | 0.259 | 0.918 | 1.102 | 1.001 | 0.959 | 1.884 | 1.020 |
| **47** | 0.794 | 0.947 | 0.786 | 0.849 | 1.683 | 0.889 |  | | |
| **48** | 0.766 | 0.829 | 0.753 | 0.766 | 1.013 | 0.587 |  |  |  |
| **Mean** | 0.967 | 1.528 | 1.017 | 0.934 | 1.482 | 0.964 | 1.032 | 2.250 | 1.188 |
| **SD** | 0.197 | 0.935 | 0.341 | 0.201 | 0.658 | 0.324 | 0.185 | 2.547 | 0.321 |

**Supplementary Table 4.** CD300e expression on myeloid cells

| **Study no** | **Baseline** | | | **Up-dosing** | | | **Maintenance** | | |
| --- | --- | --- | --- | --- | --- | --- | --- | --- | --- |
|  | **Eosinophils** | **Monocytes** | **Neutrophils** | **Eosinophils** | **Monocytes** | **Neutrophils** | **Eosinophils** | **Monocytes** | **Neutrophils** |
| **1** | 1.225 | 3.989 | 1.272 | 1.236 | 2.530 | 1.513 | 0.909 | 1.638 | 0.913 |
| **5** | 1.157 | 1.712 | 1.110 | 1.185 | 1.817 | 1.495 | 1.030 | 1.926 | 1.050 |
| **6** | 1.176 | 1.895 | 1.213 | 1.039 | 2.109 | 1.063 | 1.005 | 2.533 | 0.928 |
| **7** | 1.077 | 2.460 | 1.164 | 3.335 | 6.429 | 3.313 | 1.134 | 2.290 | 1.224 |
| **8** | 1.135 | 3.711 | 1.181 | 1.104 | 1.184 | 1.712 |  | | |
| **9** | 1.234 | 2.519 | 1.380 | 1.003 | 1.644 | 0.993 | 1.031 | 1.979 | 1.070 |
| **10** | 1.132 | 1.171 | 1.064 | 0.987 | 1.626 | 0.931 | 1.053 | 2.021 | 1.057 |
| **17** | 0.990 | 1.797 | 0.968 | 1.192 | 1.831 | 1.802 | 0.903 | 2.053 | 1.035 |
| **18** | 1.458 | 2.907 | 1.711 | 1.459 | 4.022 | 1.904 | 1.089 | 2.217 | 1.147 |
| **19** | 1.026 | 1.711 | 1.143 | 0.907 | 1.948 | 1.018 | 0.990 | 1.737 | 1.015 |
| **20** | 0.793 | 1.259 | 0.932 | 0.620 | 1.335 | 0.756 | 1.373 | 2.395 | 1.450 |
| **21** | 1.101 | 2.342 | 1.135 | 1.187 | 4.531 | 1.296 | 1.008 | 2.014 | 1.087 |
| **22** | 1.016 | 2.040 | 1.116 | 1.030 | 3.757 | 1.273 | 1.180 | 1.997 | 0.927 |
| **23** | 1.124 | 2.376 | 1.173 | 1.136 | 2.205 | 0.841 | 1.138 | 2.826 | 1.199 |
| **24** | 1.228 | 1.804 | 1.273 | 1.178 | 2.219 | 0.953 |  | | |
| **25** | 0.966 | 2.523 | 0.946 | 1.237 | 2.672 | 1.205 |  |  |  |
| **26** | 0.968 | 1.958 | 0.956 | 0.967 | 1.691 | 0.849 |  |  |  |
| **27** | 1.007 | 2.331 | 1.019 | 1.053 | 2.511 | 1.115 |  |  |  |
| **32** | 0.960 | 2.731 | 0.889 | 1.402 | 3.544 | 1.534 |  |  |  |
| **33** | 0.929 | 2.411 | 0.935 | 1.249 | 1.982 | 0.974 |  |  |  |
| **34** | 0.823 | 1.777 | 0.836 | 1.090 | 2.629 | 1.086 |  |  |  |
| **35** | 1.004 | 2.307 | 0.998 | 1.005 | 2.162 | 0.966 | 0.896 | 1.271 | 0.704 |
| **36** | 1.066 | 2.555 | 1.053 | 1.111 | 2.902 | 0.125 | 1.086 | 2.499 | 1.163 |
| **37** | 1.016 | 1.763 | 0.993 | 1.126 | 2.634 | 1.100 |  | | |
| **38** | 2.465 | 4.509 | 1.657 | 0.951 | 1.558 | 0.934 |  |  |  |
| **39** | 1.162 | 2.981 | 0.892 | 1.564 | 3.130 | 1.457 |  |  |  |
| **40** | 1.022 | 1.189 | 0.678 | 1.636 | 2.549 | 1.530 |  |  |  |
| **41** | 1.235 | 2.218 | 1.279 | 0.989 | 1.850 | 0.993 | 1.130 | 1.516 | 0.635 |
| **42** | 0.993 | 1.860 | 1.013 | 1.121 | 2.099 | 1.035 | 1.057 | 1.905 |  |
| **43** | 0.965 | 2.609 | 1.110 | 1.198 | 2.241 | 1.306 |  | | |
| **44** | 1.135 | 2.228 | 0.994 | 1.259 | 2.738 | 1.346 | 1.657 | 3.064 | 1.239 |
| **45** | 1.532 | 2.261 | 1.324 | 0.990 | 1.944 | 1.071 | 1.018 | 1.864 | 0.981 |
| **46** | 1.070 | 1.714 | 0.842 | 1.084 | 1.977 | 1.158 | 1.453 | 2.528 | 1.442 |
| **47** | 1.510 | 3.069 | 1.458 | 1.263 | 2.681 | 1.243 |  | | |
| **48** | 1.533 | 2.793 | 1.543 | 0.888 | 1.906 | 0.928 |  |  |  |
| **Mean** | 1.128 | 2.278 | 1.118 | 1.172 | 2.418 | 1.199 | 1.107 | 2.114 | 1.067 |
| **SD** | 0.274 | 0.693 | 0.215 | 0.393 | 0.949 | 0.463 | 0.190 | 0.439 | 0.207 |
